# Human-In-The-Loop Assessment of an Ultralight, Low-Cost Body Posture Tracking Device

**DOI:** 10.3390/s20030890

**Published:** 2020-02-07

**Authors:** Marek Sierotowicz, Mathilde Connan, Claudio Castellini

**Affiliations:** Institute of Robotics and Mechatronics, German Aerospace Center (DLR), 82234 Weßling, Germany; mathilde.connan@dlr.de (M.C.); claudio.castellini@dlr.de (C.C.)

**Keywords:** wearable sensors, inertial measurement units, low-cost sensors, assistive robotics, rehabilitation robotics, teleoperation, space robotics

## Abstract

In rehabilitation, assistive and space robotics, the capability to track the body posture of a user in real time is highly desirable. In more specific cases, such as teleoperated extra-vehicular activity, prosthetics and home service robotics, the ideal posture-tracking device must also be wearable, light and low-power, while still enforcing the best possible accuracy. Additionally, the device must be targeted at effective human-machine interaction. In this paper, we present and test such a device based upon commercial inertial measurement units: it weighs 575 g in total, lasts up to 10.5 h of continual operation, can be donned and doffed in under a minute and costs less than 290 EUR. We assess the attainable performance in terms of error in an online trajectory-tracking task in Virtual Reality using the device through an experiment involving 10 subjects, showing that an average user can attain a precision of 0.66 cm during a static precision task and 6.33 cm while tracking a moving trajectory, when tested in the full peri-personal space of a user.

## 1. Introduction

Multi-modal intent detection is the problem of detecting a person’s intention to move or to activate one’s muscles using sensors pertaining to different modalities, for example, going beyond the traditional usage of surface electromyography (sEMG) [1,2]. Especially in (upper-limb) prosthetics, sEMG is in use since the 50s to convert muscle activation signals, detected through the tiny electrical fields generated by motor units while contracting [3,4], into control commands for a prosthetic device. Although no clear substitute for sEMG is in sight, this technique suffers from a number of drawbacks (see, e.g., Reference [5]) and alternative means are being studied [2,6] to detect muscle activation in a different way, for example, force myography through muscle bulging [7,8] and ultrasound scanning through musculoskeletal internal movement detection.

An interesting alternative to these techniques consists of using some form of motion tracking and/or body posture detection to provide information about the body kinematics of the user, rather than directly detecting their intention to activate their muscular system. Knowing, for example, that the user is drawing their arms close to each other might be useful to enforce a coordinated two-handed prosthetic grasping of a heavy basket—this idea already appears in Reference [1]. As well, such information could be extremely valuable in solving the limb-position effect [9,10], which refers to the change in muscular recruitment and activity (and, by extension, measurable muscular signals) for the same hand movement due to a change in body pose.

Body tracking could also provide useful data for day-to-day health monitoring, besides medically relevant data for patients using prosthetic devices. With the recent interest in Internet of Things (IoT) solutions in this direction, many devices have been presented as a tool to gather this sort of information. In many cases, authors propose solutions based on optical body tracking system [11]. 

However, optical, magnetic or laser-based motion tracking are enforced by detecting the position of body markers with respect to an external source of radiation (near-infrared cameras, magnetic field generators, etc.), implying that such systems cannot be wearable – one can definitely wear the markers but not the sources of energy. Furthermore, an optical system, even if used in a wearable configuration in association with markers, will always be prone to the issue of line of sight occlusions, which is especially problematic in real time applications and when analyzing activities entailing complex body postures. As opposed to such techniques and if the user’s absolute position is not required, a potentially better alternative is represented by Inertial Measurement Units (IMUs). An IMU is in general able to detect its own acceleration and orientation with respect to the gravitational field; using a constellation of several such devices in specific spots on the human body, one can therefore reconstruct its kinematic configuration. This solution is already used, for example, in the XSens Suit (https://www.xsens.com) or in the rehabilitation device called Riablo (https://www.corehab.it/en). One well-known drawback of IMUs is that their measurements tend to drift over time, needing frequent recalibration; but with the advent of virtually drift-free IMUs-on-a-chip (e.g., Bosch’s BNO055 [12]) this problem can be overcome even using off-the-shelf components and the optimized data-fusion algorithms that come with them. The BNO055 also has the advantage of being cheap, lightweight and low-power, therefore perfect to be coupled to a wireless/Bluetooth (BT) transmission system and a small battery.

Putting all these pieces together, in this paper we introduce the BodyRig a complete, ultralight, low-cost upper body posture detector based upon such commercial IMUs. The BodyRig, consisting of a constellation of up to 7 IMU/BT/battery modules, weighs in total 575 g, it lasts 10.5 h of continual operation, it is donned and doffed in less than a minute and costs less than 290 EUR. For comparison, the XSens Awinda setup, while consisting of a more complex constellation of 17 trackers and allowing for a faster update rate (1 kHz internal, 240 Hz output rate), has a battery life of around 6 h and requires a setup procedure of 10 min [13]. 

The BodyRig can be easily used to enforce real-time body posture tracking, teleoperation and multi-modal prosthetic control, both in Virtual Reality (VR) and in real life. We here assess the device through an experiment involving 10 users in an online trajectory-tracking task in VR, showing that it affords an average precision of 0.66 cm during a precision task and 6.33 cm while tracking a target moving along a prerecorded trajectory, when tested in the full peri-personal space of a user. These values are much larger than the absolute precision of the VR system employed in the experiment, which appears reasonable, given the pointing precision attainable by a human being [14].

Potential applications of this device, not limited to rehabilitation robotics [15] and discussed in detail at the end of this paper, are endless.

## 2. Materials and Methods

The BodyRig system was used, in this experiment, in a reduced configuration, which allows monitoring of user movements up to and including the forearms. In this reduced configuration, the total material cost is around 220 euros and the total weight around 540 g and can operate more than 13 h uninterruptedly. In this case, the system consists of 5 independent modules, each one fundamentally consisting of an IMU (in our case, Bosch’s BNO055 sensors [12]), a BT module and a battery. The IMUs are connected via I2C to a bluefruit feather nRF52832 breakout board module from Adafruit (see References [16,17] for more details), which is capable of communicating using the Bluetooth Low Energy stack via a proprietary serial port emulation profile from Nordic Semiconductors called BLEUart (see Reference [18]). Each module acts as a peripheral within the BLEUart standard, except the one monitoring the movements of the user’s core, which acts as a central unit. The data transmitted from the peripherals to the central is relayed to a host computer via conventional Bluetooth SPP using a RN41 module from Microchip Technology (see Reference [19]).

All components are off-the-shelf, except for the 3-D printed casings and the custom PCB for the RN41 module. At the current stage, the BodyRig can be considered a minimum viable product (MVP) but expansions are planned in the near future.

**Figure 1:**
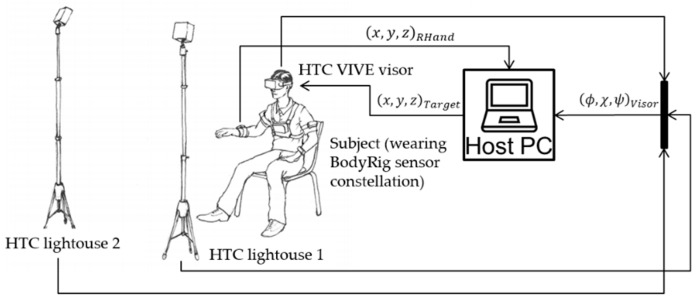
Block diagram and illustration of the setup.

The HTC Vive VR visor (see Reference [20]) was used to provide the subjects with visual feedback in virtual reality. The visor uses two light sources to determine its own orientation and position, although in this experiment only the orientation of the visor was needed. The precision of the Vive system, measured using a “tracker,” is sub-millimetric in a static configuration [21,22]. The position of the avatar’s head was determined using forward kinematics based on the data coming in from the BodyRig system together with the orientation of the head as measured by the visor itself. The BodyRig monitored the orientation of trunk, both humeri and both forearms. Therefore, considering the measurement of the user’s head’s orientation with the visor, a total of 6 body segments were monitored. Figure 1 shows a diagram with the fundamental elements of the setup.

The data from each BodyRig module is asynchronously sent to the host PC with an average frequency of about 75 Hz. Considering that the kinematic model is updated every time data from one peripheral is transmitted to the host PC, in order to handle this transmission frequency from 5 trackers, the main software acquires the incoming data at 400 Hz. The data relative to the user’s posture is sent from the application managing the serial driver to the VR simulation together with the target’s position. Each target movement profile is the result of a recording obtained using the BodyRig in an analogous configuration as the one used during the experiment.

Within the simulation, the position and orientation of the body segment of the avatar are represented based on the measurements of the BodyRig, according to a forward kinematic model (please refer to Reference [23] for more details). The lengths of the avatar’s body segments were the same for all subjects. These lengths are important, as the position of the avatar’s right hand in space is a function of these parameters. The body segment lengths are indicated in Table 1.

Ten male, right handed subjects of age 29.1 ± 7.2 years, weight 73.7 ± 8.1 kg and height 1.82 ± 0.07 m took part in the experiment. The experimental procedure was thoroughly explained to the subjects in both oral and written form prior to the experiment and all the participants signed an informed consent form. The experiment was performed according to the World Medical Association’s Declaration of Helsinki, regarding the ethical principles for medical research involving human subjects, last version, as approved at the 64th WMA General Assembly, Fortaleza, Brazil, October 2013. Data collection from subjects was formally approved by the host institution’s internal board for protection of data privacy and by the work council of the German Aerospace Center. A physician is part of the council that approved the experiments.

**Figure 2:**
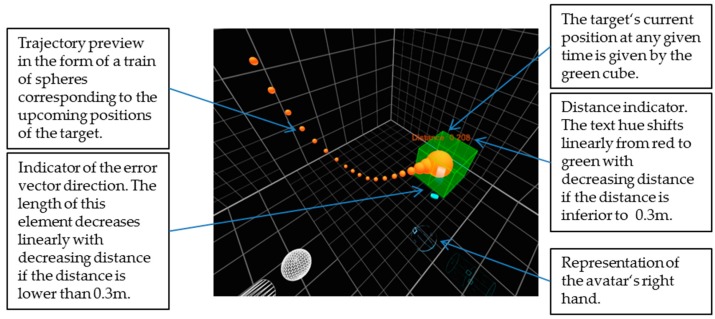
A representative screenshot of the Virtual Reality (VR) simulation with description of each element.

In VR, the subjects were provided with a 3D representation of their avatar, the current position of the target and a train of spheres marking the upcoming positions of the target along its trajectory. Additionally, the subjects were provided with a textual indicator of the current distance of the target from the avatar’s right hand. Furthermore, a cylinder was made visible in VR to the users, which from the center of the target pointed at the right hand of the avatar, providing a useful indication of the direction along which it was necessary to correct for the current error at each given time. Figure 2 depicts a more detailed description of each relevant element present in the VR simulation.

During the experiment, the data recorded included the position and orientation of each body segment monitored by the BodyRig, the position of the target and of each individual sphere from the train of spheres used in the simulation to inform the subject about the upcoming trajectory of the target. Each subject was asked to follow the target along 3 distinct trajectories, prerecorded using the BodyRig, at 2 different speeds, with 3 repetitions per speed and trajectory, for a total of 18 trials. The trajectories are hereafter respectively indicated as Infinity-shaped trajectory (IS), Constant speed Tai Chi (CT) and Variable speed Tai Chi (VT). No randomization in the order of execution of the target profiles was employed, as verifying whether better performance is achievable with a particular profile rather than another is not the goal of this experiment.

In order to familiarize themselves with the VR environment and with the equipment, the subjects were first asked to follow a separate trajectory, especially recorded to serve as a familiarization tool, at 2 different speeds with 1 repetition per speed. The familiarization target trajectory is hereafter indicated as Familiarization Profile (FP).

Between each of the 4 sets of trials with a common target trajectory (including the familiarization phase) the subjects were asked to rest at least 60 s. During this resting time, the subjects were asked to record a subjective assessment of the perceived difficulty of the previous task, as well as the perceived influence of both their current level of fatigue and the BodyRig system on their accuracy and reactivity. The assessment was to be given through a score ranging from 1 to 20. Furthermore, at the end of the experiment, the subjects were asked to fill a System Usability Scale questionnaire (see Reference [24]) in order to assess the perceived practicality of the BodyRig system in the context of the tasks at hand.

**Figure 3:**
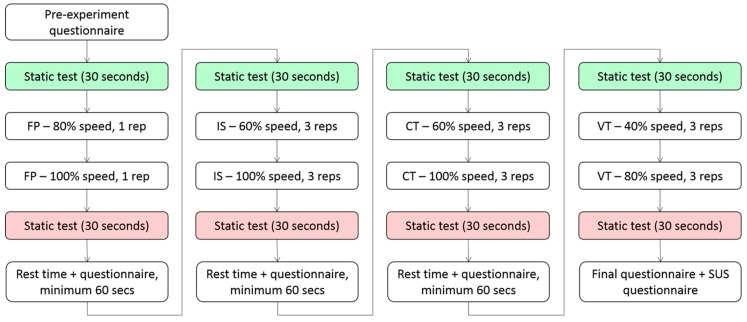
Flowchart illustrating the experiment’s execution. The green and red color represent the static tests executed after the resting phases and after completion of a task series, respectively.

Before and after each of the 4 sets of trials with a common target trajectory, the subjects were asked, for 30 s, to keep the avatar’s right hand as close as possible to a static point located in the frontal region of the peri-personal space, reachable by keeping the right arm in an almost extended pose. Figure 3 shows a flow chart of the main phases of the experiment’s execution. Additional details regarding the BodyRig system and the experiment execution can be found in the supplementary material attached to this article (see video S1 in Supplementary Materials). 

## 3. Results

Data analysis was based on properties measured both from the trajectories followed by the test subjects, as well as from the target trajectories themselves. 

### 3.1. Static Tracking

**Figure 4:**
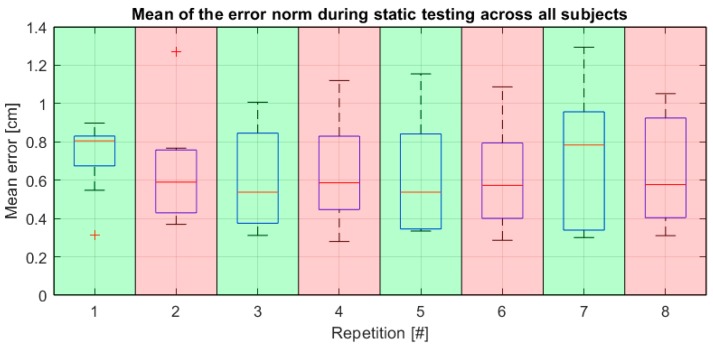
Boxplot over all subjects of the mean error norm measured over each static testing session.

**Figure 5:**
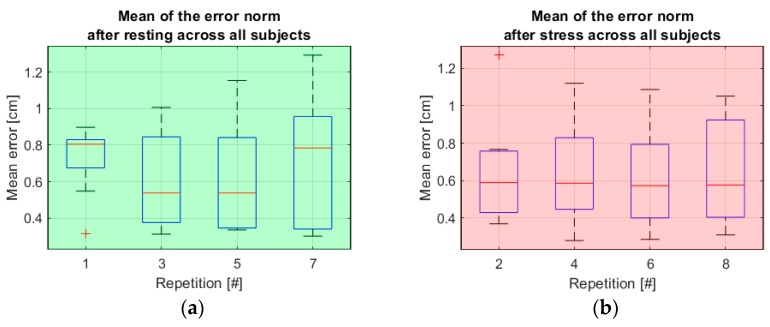
Boxplots of the error during static tests specifically after resting phases and after completion of one task series. (**a**) Boxplot of the mean error in static tests executed after a resting phase; (**b**) Boxplot of the mean error in static tests executed after completion of a task series, before a resting phase.

During the static tests, the subjects were asked to keep the sphere representing the avatar’s right hand as close as possible to a static target for 30 s. As a general measure of the subject’s precision, the mean and the variance of the norm of the vector difference between the end of the avatar’s right forearm and the target’s position was drawn over the course of the 30 s of each trial, in an analogous way as shown in Equation (7). One of the subjects provided strongly and consistently outlying data: the mean of the error during static tests from this subject shows as the only outlier among the data from all subjects for 7 static tests out of 8, according to the generalized extreme Studentized deviate test for outliers. After qualitative analysis, it seems that this is either due to a failure, on the part of the subject, to understand the provided indications and to utilize the feedback from the VR simulation properly in order to correct the position of the right hand or possibly due to technical problems. However, no such anomaly has been observed with any other subject. For this reason, the data gathered during static tests for this subject were not considered in the analyses. Considering all remaining subjects and repetitions, the two metrics have values of 0.66 ± 0.27 cm for the mean and 0.0835 ± 0.0744 cm^2^ for the variance, respectively. These metrics present a correlation coefficient of 0.63 with a *p* < 0.0001. Figure 4 shows a boxplot of the error measured during static tests over all reliable subjects sorted by test number. Figure 5 shows boxplots of the mean static error as observed specifically after a resting phase and after completion of a full task series, with the intent of showing the behavior of the error in these two specific conditions. In both cases, the data is color-coded consistently with Figure 3.

### 3.2. Trajectory Tracking

For the purposes of this analysis, the recorded positions of the avatar’s right hand were synchronized with the respective target’s position recordings and both trajectories were synchronized across subjects and repetitions using a uniform sampling time as basis for the synchronization.

Furthermore, for each trajectory point, the average difference vector between the hand’s position and the target’s position was divided in its parallel and orthogonal components with respect to the local tangential vector of the target’s trajectory, so that the following relation applies:

(1) $$\bar{\;e\;}\left(k\right)\;=\sqrt{e_{∥}{\left(k\right)}^{2}+e_{⊥}{\left(k\right)}^{2}}$$

where $\bar{\;e\;}\left(k\right)\;$ is the average norm of the error vectors $\vec{\;e\;}$ from all subjects and repetitions, whereas $e_{∥}\left(k\right)$ and $e_{⊥}\left(k\right)$ are the average norms of the components of $\vec{\;e\;}\vec{\;e\;}$ that are respectively parallel and perpendicular to the tangent direction of the target’s trajectory at the k-th sample from the start of the trial, for which the average error is computed. Furthermore, between the k-th sample of the tangent direction ${\vec{\;t\;}}_{\mathrm{trgt}}\left(k\right)$ of the target’s trajectory, the k-th sample of the target’s speed vector ${\vec{\;v\;}}_{\mathrm{trgt}}\left(k\right)$ and its norm $v_{\mathrm{trgt}}\left(k\right)$, the following relations exist:

(2) $${\vec{\;v\;}}_{\mathrm{trgt}}\left(k\right)=\;\frac{\left({\vec{\;p\;}}_{\mathrm{trgt}}\left(k+1\right)-{\vec{\;p\;}}_{\mathrm{trgt}}\left(k\right)\right)}{t\left(k+1\right)-t\left(k\right)},$$

(3) $$v_{\mathrm{trgt}}\left(k\right)=∥{\vec{\;v\;}}_{\mathrm{trgt}}\left(k\right)∥$$

(4)  t →trgt(k)=  v →trgt(k)vtrgt(k),

where ${\vec{\;p\;}}_{\mathrm{trgt}}\left(k\right)$ is the k-th sample of the target’s position and t(k) is the timestamp corresponding to the k-th sample. Furthermore, the k-th sample of the target’s acceleration vector ${\vec{\;a\;}}_{\mathrm{trgt}}\left(k\right)$ and its norm $a_{\mathrm{trgt}}\left(k\right)$ are computed according to

(5) $${\vec{\;a\;}}_{\mathrm{trgt}}\left(k\right)=\;\frac{\left({\vec{\;v\;}}_{\mathrm{trgt}}\left(k+1\right)-{\vec{\;v\;}}_{\mathrm{trgt}}\left(k\right)\right)}{t\left(k+1\right)-t\left(k\right)},$$

(6) $$a_{\mathrm{trgt}}\left(k\right)=∥{\vec{\;a\;}}_{\mathrm{trgt}}\left(k\right)∥.$$

Additionally, both the velocity and the acceleration vectors were filtered through a moving average filter in order to increase the numerical stability for the purpose of statistical analysis. The width of the moving window is 0.2 s. 

In all cases, the complete series of samples of a property p corresponding to a given task T is hereinafter indicated with $p^{T}$ or simply p when indicating the concatenation of all samples corresponding to all tasks.

**Figure 6:**
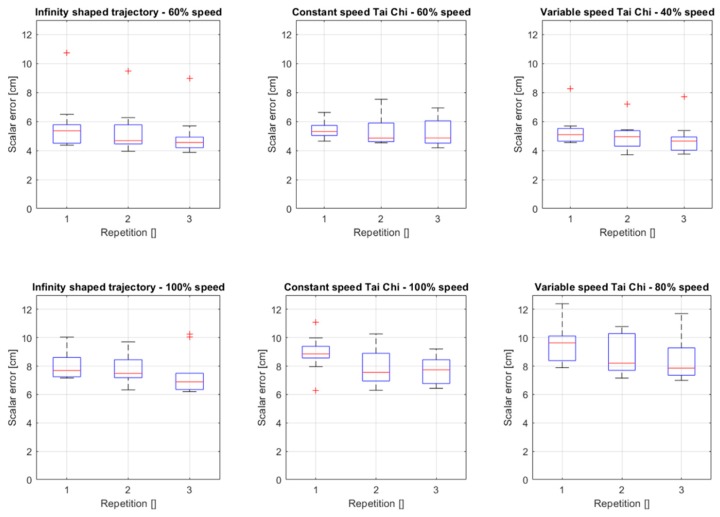
Boxplots of the scalar error over the repetitions for each target profile and speed.

Considering the average performance over all subjects, we can observe the trend of the scalar error over each repetition. The results are shown in Figure 6. The performance, in this case, is measured by the mean scalar error over all $N_{\mathrm{seq}}$ samples acquired during a single sequence

(7) $${\bar{\;e\;}}_{\mathrm{rep}}\;=\frac{1}{N_{\mathrm{seq}}}\sum_{1\leq k\leq N_{\mathrm{seq}}}^{}e_{\mathrm{rep}}\left(k\right),$$

and the properties shown in the boxplots are computed over the performance of the 10 subjects and sorted by repetition number.

Observing the average performance over all subjects and over all repetitions, we were able to measure general relations between the error metrics and the movements of the subjects.

The Pearson’s correlation coefficients among the main properties of the target trajectory and the error metrics are reported in Table 2. 

The average trajectory followed by the test participants is computed across all repetitions and subjects and the correlation coefficients are computed for the entirety of the samples. The k-th sample of the average error is computed according to

(8) $$\bar{\;e\;}\left(k\right)\;=\frac{1}{N_{\mathrm{rep}}N_{\mathrm{subj}}}\sum_{1\leq \mathrm{sub}\leq N_{\mathrm{subj}}}^{}\sum_{1\leq \mathrm{rep}\leq N_{\mathrm{reps}}}^{}e_{\mathrm{sub},\mathrm{rep}}\left(k\right),$$

where $e_{\mathrm{sub},\mathrm{rep}}\left(k\right)$ is the k-th sample of the scalar error measured for subject sub and repetition rep.

The means and variances of the error metrics are reported in Table A1.

**Figure A1:**
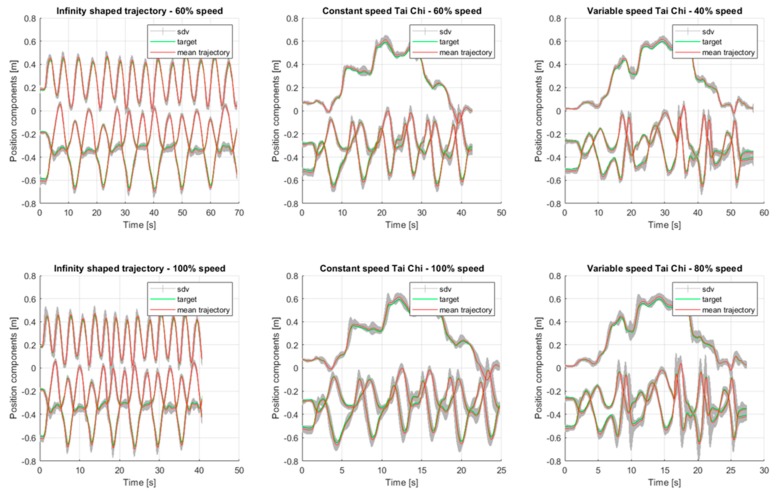
Average over all subjects and repetitions with standard deviation and target position.

**Figure A2:**
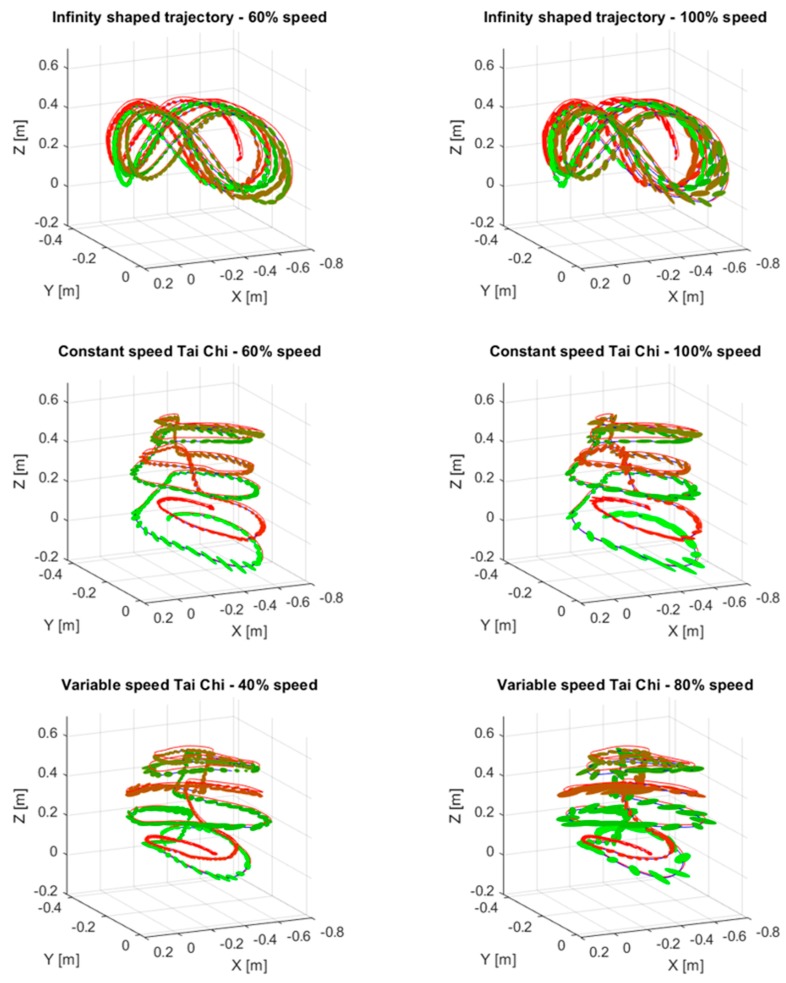
Trajectory with error ellipsoids. The color shifts from red to green over time during target’s movement.

Additionally, visual inspection of the error was operated. To this end, a plot with the single components of the target trajectory and the standard deviation of the error by all subjects in all repetitions was produced (see Figure A1). Furthermore, visual inspection was operated by plotting, in the form of ellipsoids, the covariance matrix corresponding to a subset of points lying on the target trajectory of each dynamic tracking task. The results are shown in Figure A2. The covariance matrix is computed according to

(9) $$\Sigma \left(k\right)\;=\frac{1}{N_{\mathrm{rep}}N_{\mathrm{subj}}-1}E{\left(k\right)}^{T}E\left(k\right),$$

where E(k) is the 30 × 3 error observation matrix, with each row representing the k-th three dimensional error vector $\vec{\;e\;}\left(k\right)$ measured from a specific subject and repetition. The plots used for visual inspection are shown in Figure A1 and Figure A2.

### 3.3. Subjective Assessment

As mentioned above, the subjects were asked to provide a subjective assessment of several relevant factors by assigning a score ranging from 1 to 20. In this context, accuracy is to be understood as the capability of maintaining the position of the right hand within the smallest possible margin of a given static target position in virtual reality and reactivity as the capability to quickly react to sudden changes in direction and speed of the target. All the subjects were provided with the aforementioned definitions.

Assuming that all subjects operated on a relative but self-consistent scale, the average of each score has been drawn across all subjects, whereas the variance of these scores was ignored. The average scores are reported in Table 3. 

Looking at the results from the System Usability Scale questionnaire [24], the subjects assigned an average score of 85 points to the system, corresponding to an A for the general usability of the device. 

The subjective assessments provided by the subjects after the completion of each set of repetitions associated with a specific target trajectory profile seems to consistently bear no significant correlation to the objective error metrics. This could indicate that, independently from the subject’s perception of the hardness of a task, all subjects were able to achieve it with uniformly low error.

## 4. Discussion

### 4.1. General Discussion

From the average scores assigned by the subjects, it is clear that, on average, the difficulty of the task was perceived as being consistently increasing. However, while the tasks involving the profiles IS and CT were considered at almost the same level of difficulty, the tasks involving IS were considered as more physically demanding in terms of the influence of fatigue on accuracy (see row 2 on Table 3).

The data from the static trials give a general measure of the average achievable precision in terms of average distance from the desired point. These metrics appear to be highly correlated, which seems to indicate that inaccuracy in maintaining a given position manifests itself consistently in both the inability of exactly reaching the desired position and in the instability of the hand’s position. Possible sources of error in this type of trial could be fatigue, as well as non-clarity in the display of the magnitude and direction of the difference vector between the target’s center and the avatar’s hand. Observing the metrics over all the subjects as a function of the repetition number (see boxplots in Figure 4), no obvious influence from fatigue is evident and there is only a slight improvement visible between the very first and the following repetitions, due most likely to learning effect. The absolute precision of the Vive tracking system has been assessed as sub-millimetric in a static configuration [21,22] and, reasonably, the positioning accuracy of the target in VR was in the same range. Given this fact, the average tracking precision of 66mm obtained by the subjects seems appropriate. The human precision in pointing has actually already been determined to be about half a centimeter in Reference [14]—for further details, see also the references therein.

However, some noteworthy behavior is observable looking at the error in static tests specifically after the resting phases and after the completion of a task series (see Figure 5). Namely, one can notice, in the case of static tests executed immediately after completion of a series, that the error remains largely consistent after repetition number 2. Conversely, the error maxima seem to increase consistently in the case of static tests executed after a resting phase. This could be dictated, for the first case, by the musculature being more tonic and responsive immediately after prolonged but moderate stress and the subjects being on average more focused on the accuracy of their movements. On the other hand, right after the resting phases, during which a questionnaire was to be filled, the focus of the subjects may have been deviated from movement coordination. Fatigue is, of course, the most likely cause for the inconsistency in the mean static error observed after resting phases.

During the tracking of a dynamically moving target, the component of the error tangential to the target’s trajectory presents a higher correlation with the total average error magnitude than the orthogonal component (see Table 2). However, the average magnitude of the parallel error component is not far greater than the average magnitude of the orthogonal error component (see Table A1).

Furthermore, it is worth mentioning the presence of a high correlation between the target’s velocity and the error’s magnitude and specifically between the target’s velocity and the error’s component parallel to the trajectory. This would seem to indicate that the most consistent source of error is the subjects not being able to follow the target’s exact position onto its trajectory, rather than being able to maintain a short distance from the trajectory’s path itself. The average orthogonal component seems to be less consistently present in the final average error norm and does not show as high a correlation with the target’s speed.

There is, however, a moderate correlation between the orthogonal component of the scalar error and the target’s acceleration. This is likely because, typically, most subjects find it difficult to follow the target accurately when this makes a sudden change in direction and the error in this case has a strong component perpendicular to the target’s trajectory.

Lastly, Figure A1 and Figure A2 provide a further qualitative analysis of the trajectories, showing in three dimensions the deviation from the target trajectory along the different target profiles. Figure A2, in particular, shows the error ellipsoids along the target trajectory, obtained by computing the covariance matrix from all the measured error vectors across all subjects and repetitions (see Equation (9)). The color of the depicted ellipsoids transitions from red to green over time during the target’s movement. From Figure A1 one can notice an increased standard deviation when the target moves faster and when it suddenly changes direction. From Figure A2 it is apparent that the error vector components dramatically increase both as the target gets faster and as the subjects approach the points of maximum curvature of the trajectories.

Interestingly, it seems that the correlation between the perpendicular component of the error and the norm of the acceleration vector consistently increases with the successive target trajectory profiles (see Table A1). This could indicate the effect of fatigue coupled with an increasing rate of perceived difficulty (see Table 3), which renders the subjects progressively less effective at following sudden changes in direction of the target, which themselves become more pronounced during later target profiles.

The correlation coefficient between average orthogonal error component and acceleration norm corresponding to the IS tasks at 60% speed is noticeably much lower than all the following ones. A lower correlation in this case is to be expected because of the repetitiveness of this particular task. Due to this factor, it is possible that, especially after a few cycles, the subjects would start to execute the same action cyclically, rather than actively follow the target and this would lead to low correlation between the target’s motion and the error’s components. It is possible that central pattern generators [25] played a role in this particular task. However, this should also happen for the IS tasks at 100% speed. The most likely explanation for this is that, with the target moving faster, the subjects would naturally follow it more actively.

### 4.2. Limitations

It should be noted that the goal of this study is not to validate the absolute accuracy and precision of the BodyRig system, for whose task an absolute reference would be needed, for instance a VICON marker; but rather to assess the achievable performance in a range of tasks as wide as possible. In this study, the role of the ground truth is played by the target, rather than by an optical system used as reference. Any validation of the accuracy of the BodyRig in absolute terms should of course make use of a parallel body tracking setup for reference.

The BodyRig uses a kinematic model to determine the positions of the body segments and therefore it does not measure these positions directly. If the application requires exact absolute position determination, the BodyRig is prone to errors due to mismatch between the link length of the kinematic model and of the user’s body.

Relevant applications which would require validation of the BodyRig in absolute terms are, for example, the measurement of anthropometric data in Cartesian space, such as the measurement of the extension of a subject’s useful dexterity space. In such applications, the subject’s proportions would have to be fed into the kinematic model and validation against an absolute reference would be needed to assess accuracy of the measurements. However, the BodyRig does provide useful kinematic data, such as kinematic angles, without the need for any reference or measurement of the user’s body proportions. These angular measurements should have resolution and accuracy compliant with the specifications in Reference [12,26]. Such data can be used in multi-modal intent detection and makes the BodyRig a useful input device in VR and telemanipulation applications.

This experiment does show that good performance is achievable even without fitting the kinematic model to the user’s actual body proportions, as the subjects of the study proved able to naturally and intuitively follow the target in VR. 

## 5. Conclusions

In this paper, we presented the results of a user study with the main objective of evaluating the attainable precision using the BodyRig upper body tracking system in the achievement of various tasks, as well as providing a system usability score [24]. The device allows to adjust the workspace dimensions, allowing a user to be more precise at the cost of the reduction of the workspace size or vice versa. Therefore, the measurements we operated are contingent to the specific sizes chosen for the body segments of the avatar. That said, we demonstrated that an average user can achieve a precision inferior to the centimeter in static conditions and of about 6 cm when following a target, even when this is moving at fairly rapid speeds. 

Additionally, we analyzed the effects of adaptation to a particular target movement profile in terms of mean error and standard deviation. 

Furthermore, we were able to draw distinctions between the main error sources in following the target’s movements. We were able to link statistically the error component parallel to the target’s trajectory with the target’s speed and the error component orthogonal to the target trajectory with the target’s acceleration.

## Figures and Tables

**Table 1:** Avatar body segment lengths in centimeters.

Body Segment	Length [cm]
Thorax (Pelvis to neck base)	25
Shoulder (neck base to proximal humerus, right and left)	25
Humerus (right and left)	25
Forearm (right and left)	30

**Table 2:** Pearson correlation coefficients among average properties of movement over all tasks. Below the diagonal are the Pearson’s cross correlation coefficients. In all cases, the coefficients are statistically significant with p far smaller than 0.0001.

	p	$e_{∥}$	$e_{⊥}$	$v_{\mathrm{trgt}}$	$a_{\mathrm{trgt}}$
c					
$e_{∥}$					
$e_{⊥}$		0.516			
$v_{\mathrm{trgt}}$		0.752	0.538		
$a_{\mathrm{trgt}}$		0.498	0.679	0.626	
$\bar{\;e\;}$		0.948	0.751	0.750	0.619

**Table 3:** Average scores across all subjects sorted by assessed factor and target trajectory profile.

Assessment	FP	IS	CT	VT
Perceived difficulty	7.3	9.5	9.8	11.6
Perceived influence of fatigue on accuracy	4.4	9.1	8.5	8.8
Perceived influence of fatigue on reactivity	5.5	8.3	9.0	8.6
Perceived influence of BodyRig on accuracy	6.1	6.7	7.4	8.4
Perceived influence of BodyRig on reactivity	6.6	6.9	7.3	7.7

## Supplementary Materials

The following are available online at https://www.mdpi.com/1424-8220/20/3/890/s1. Video S1: description of the proposed device and of the experiment.

## Appendix A

**Table A1:** Relevant properties of target motion and average trajectory followed by the users (± standard deviation where applicable). All correlation coefficients have p coefficients far smaller than 0.0001.

Measurement [unit]	IS-60%	IS-100%	CT-60%	CT-100%	VT-40%	VT-80%	ALL
**Dynamic tests**							
$\mu \left(\left\|\vec{e}\right\|\right)$ [cm]	5.365 ± 1.405	7.935 ± 2.277	5.311 ± 1.865	8.148 ± 3.315	5.061 ± 2.954	8.904 ± 5.824	6.325 ± 3.248
$\mu \left(e_{∥}\right)$ [cm]	4.051 ± 1.698	6.766 ± 2.490	3.547 ± 1.932	6.038 ± 3.773	3.399 ± 2.891	6.398 ± 5.630	4.684 ± 3.263
$\mu \left(e_{⊥}\right)$ [cm]	3.472 ± 0.738	4.369 ± 0.954	3.842 ± 1.167	5.287 ± 1.849	3.458 ± 1.371	5.620 ± 2.852	4.066 ± 4.684
$\mu \left(\left\|\vec{v}\right\|\right)$ [cm]	23.108 ± 6.290	38.664 ± 9.101	18.666 ± 9.449	31.741 ± 14.592	15.091 ±1 1.169	30.743 ± 20.530	24.679 ± 13.957
$\mu \left(\left\|\vec{a}\right\|\right)$ [cm/s^2^]	33.236 ± 15.830	76.585 ± 30.815	32.333 ± 18.495	78.750 ± 40.265	28.313 ± 28.616	87.285 ± 80.367	48.731 ± 42.854
Corr($\left\|\vec{v}\right\|$,${\;e}_{∥}$) []	0.522	0.626	0.763	0.773	0.773	0.685	0.752
Corr(,${\left\|\vec{a}\right\|\;e}_{⊥}$) []	0.050	0.332	0.503	0.534	0.629	0.764	0.679
